# Supplementary material for: Natural antisense transcripts of MIR398 genes suppress microR398 processing and attenuate plant thermotolerance
Source: Nat Commun. 2020 Oct 22;11:5351. doi: 10.1038/s41467-020-19186-x (PMC7582911; doi:10.1038/s41467-020-19186-x)
Supplement: Supplementary file 1 — Supplementary Information [file 41467_2020_19186_MOESM1_ESM.pdf]

Supplementary information

Natural antisense transcripts of *MIR398* genes suppress microR398 processing and attenuate plant thermotolerance

Yajie Li, Xiaorong Li, Jun Yang, and Yuke He

Supplementary Figure 1. Genes flanking *MIR398* genes in *Brassica rapa*.

Supplementary Figure 2. Complementary regions between *MIR398* genes and their *cis*-NATs.

Supplementary Figure 3. Expression levels of *MIR398* genes and the temporal expression pattern of the *MIR398a* gene.

Supplementary Figure 4. Relative expression levels of genes in *MIR398b/c* and *NAT398b/c* transgenic plants.

Supplementary Figure 5. Mutant sites and gene relative expression levels in *CRNAT398b* plants.

Supplementary Figure 6. Relative expression levels of pri-miR398b and miR398 in transgenic plants.

Supplementary Figure 7. Northern blot showing miR398 accumulation in transgenic and mutant plants.

Supplementary Figure 8. ChIP analysis showing relative accumulation of RNA Pol II in *p35S::NAT398b* and *p35S::NAT398c* plants.

Supplementary Figure 9. Relative expression levels of *MIR398b/c* in Arabidopsis co-expressing *p35S::MIR398b/c* and *p35S::NAT398b/c* plants.

Supplementary Figure 10. Relative expression levels of *NAT398b* and pri-miR398b in transgenic plants.

Supplementary Figure 11. Relative expression levels of nat-siR398-1 and nat-siR398-2 in transgenic plants.

Supplementary Figure 12. Examination of nat-siR398b-1 accumulation in some small RNA biogenesis mutants.

Supplementary Figure 13. Gene relative expression levels and GUS signals under heat stress.

Supplementary Figure 14. Relative expression levels of *CSD1* in *csd1* and *p35S::CSD1* seedlings.

Supplementary Figure 15. H_2O_2 accumulation in transgenic plants.

Supplementary Figure 16. Results of a thermotolerance assay of *p35S::MIR398a*, *p35S::MIR398b*, *p35S::MIR398c* and *p35S::CSD1* plants.

Supplementary Table 1. *cis*-NATs of *MIRNA* genes.

Supplementary Table 2. Reads of miR398 obtained by sRNA-seq.

Supplementary Table 3. miR398 accumulation in *csd1*, *p35S::NAT398b* and *p35S::MIR398a* plants compared with the wild type as ascertained by sRNA-seq.

Supplementary Table 4. RACE results showing the relative accumulation of incorrect cleavage sites in 5′ single-stranded RNA regions of pri-miR398b.

Supplementary Table 5. Degradation products from pri-miR398b determined by sRNA-seq.

Supplementary Figures

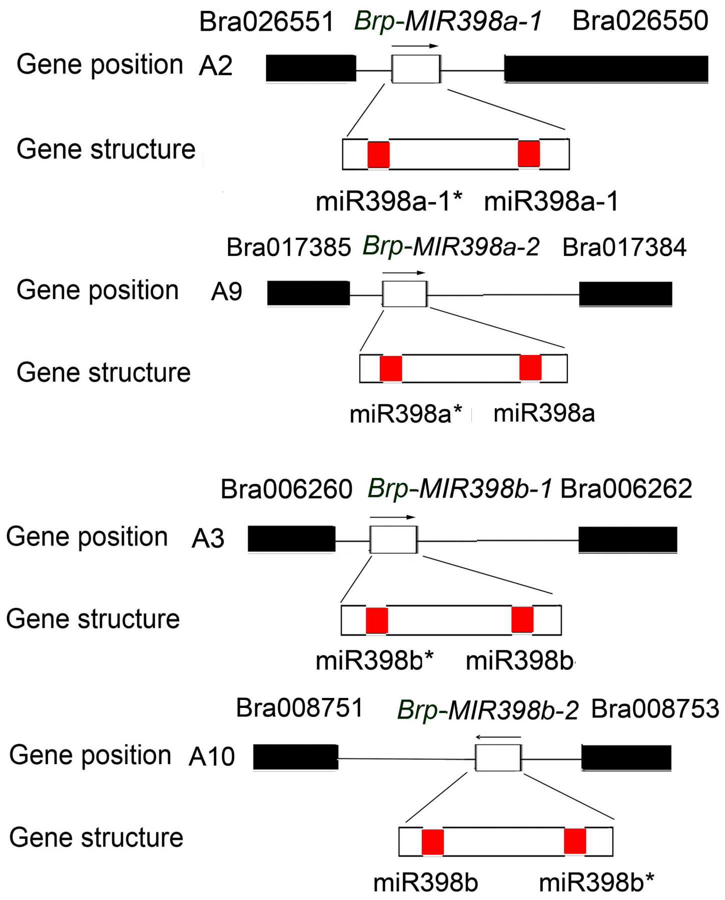

Supplementary Figure 1. Genes flanking *MIR398* genes in *Brassica rapa*. Flanking genes, *MIRNA* genes and miR398/miR398* are enclosed in black, white and red boxes, respectively. Solid lines indicate introns and intergenic regions. Arrows indicate transcription directions.

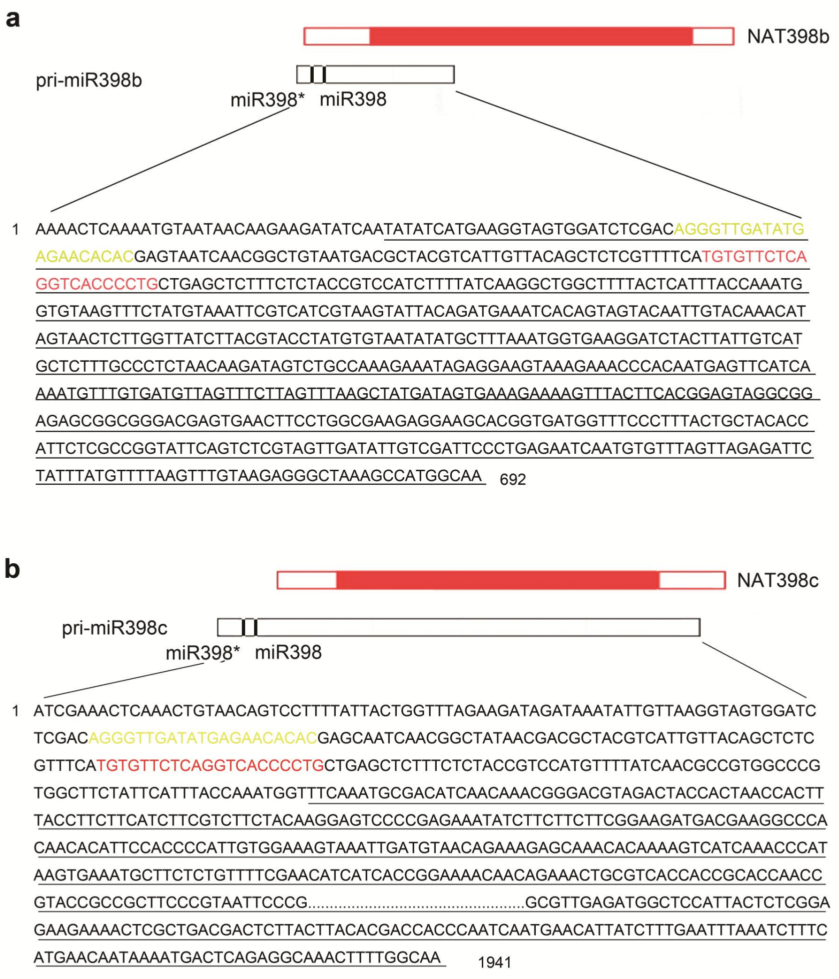

Supplementary Figure 2. Complementary regions between *MIR398* genes and their *cis*-NATs. **(a)** The reverse complementary regions between pri-miR398b and NAT398b transcript in Arabidopsis. **(b)** The reverse complementary regions between pri-miR398c and NAT398c transcript in Arabidopsis. Nucleotides in yellow indicate miRNA*, nucleotides in red indicate miRNA, nucleotides underlined indicate complementary region, dotted line indicate omitted nucleotides of pri-miR398c.

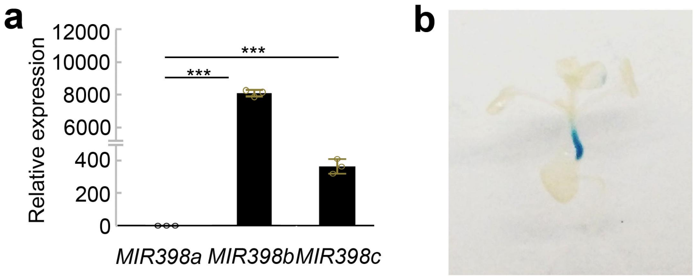

Supplementary Figure 3. Expression levels of *MIR398* genes and the temporal expression pattern of the *MIR398a* gene. **(a)** Expression levels of *MIR398a*, *MIR398b* and *MIR398c* genes in Arabidopsis. **(b)** GUS signals showing temporal and spatial expression of *MIR398a*. The experiments were repeated at least four times with similar results, and data from one representative experiment are shown. Error bars are the mean ± SD. Significant differences were determined by one-tailed student's *t*-test (*, $P < 0.05$; **, $P < 0.01$; ***, $P < 0.001$).

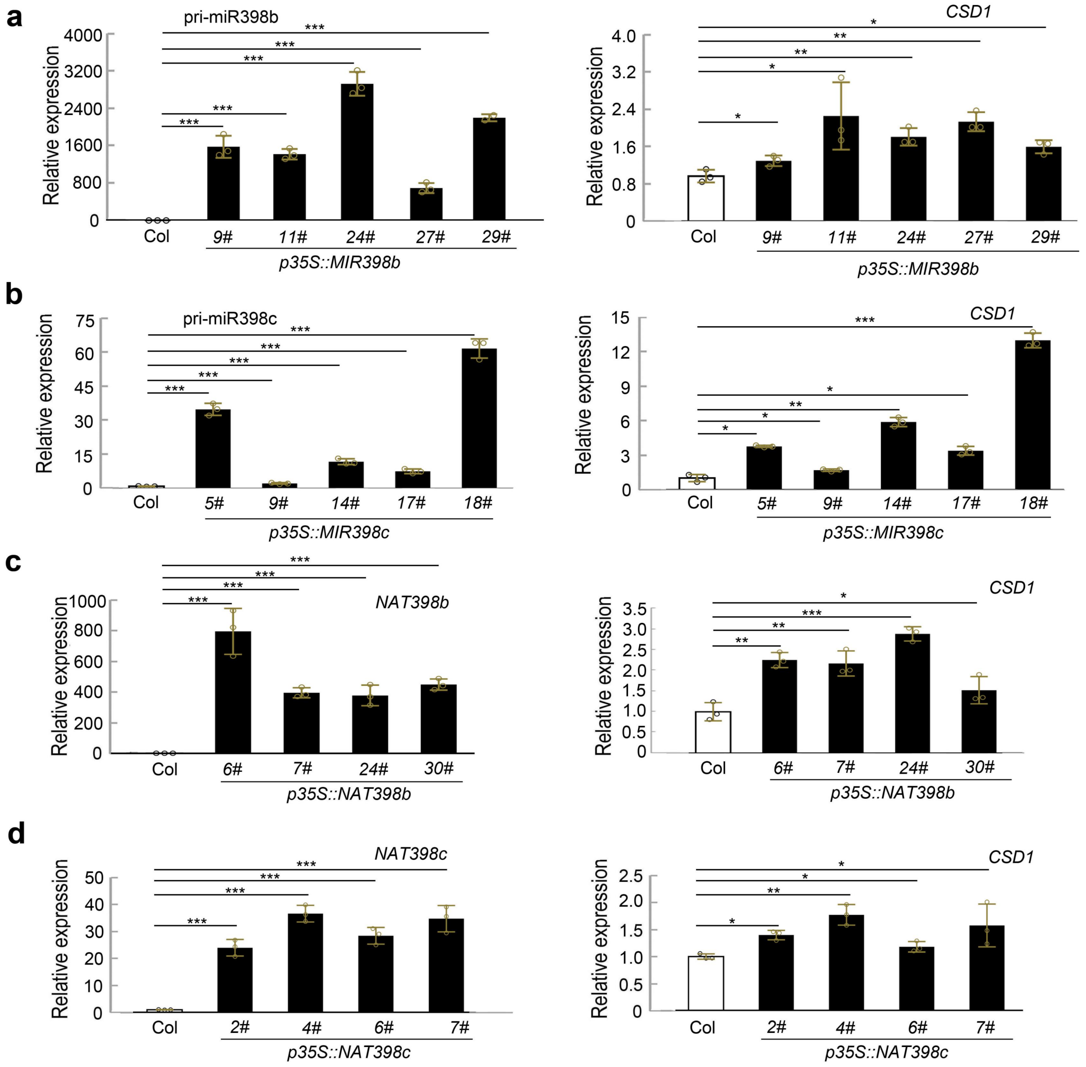

Supplementary Figure 4. Relative expression levels of genes in *MIR398b/c* and *NAT398b/c* transgenic plants. **(a–d)** qRT-PCR showing relative expression levels of the related genes in *p35S::MIR398b* **(a)**, *p35S::MIR398c* **(b)**, *p35S::NAT398b* **(c)** and *p35S::NAT398c* **(d)** plants. Error bars are the mean ± SD. Significant differences were determined by one-tailed student's *t*-test (*, $P < 0.05$; **, $P < 0.01$; ***, $P < 0.001$).

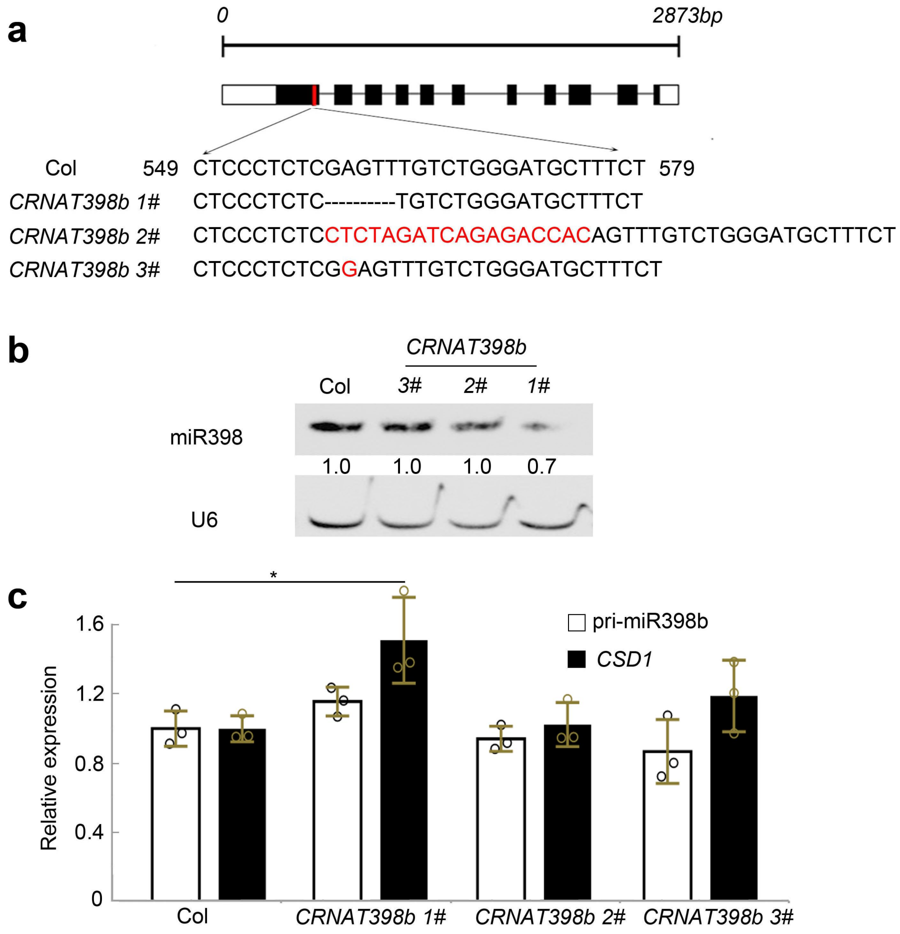

Supplementary Figure 5. Mutant sites and gene relative expression levels in *CRNAT398b* plants. **(a)** Mutant sites of *NAT398b* in *CRNAT398b* plants. **(b)** Northern blotting showing miR398 accumulation in *CRNAT398b* plants. **(c)** Relative expression levels of pri-miR398b and *CSD1* in *CRNAT398b* plants. Error bars are the mean ± SD. Significant differences were determined by one-tailed student's *t*-test (*, $P < 0.05$).

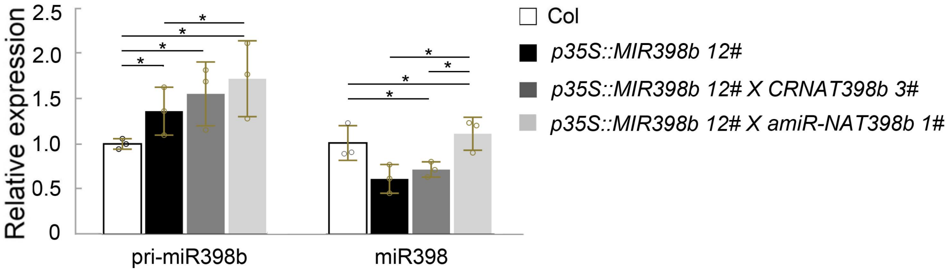

Supplementary Figure 6. Relative expression levels of pri-miR398b and miR398 in transgenic plants. Error bars are the mean ± SD. Significant differences were determined by one-tailed student's *t*-test (*, $P < 0.05$).

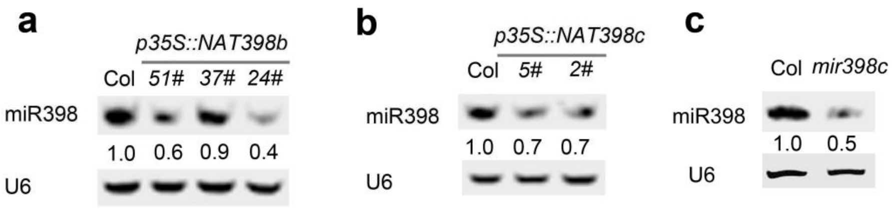

Supplementary Figure 7. Northern blot showing miR398 accumulation in transgenic and mutant plants. **(a–c)** Northern blotting showing miR398 accumulation in *p35S::NAT398b* **(a)**, *p35S::NAT398c* **(b)** and *mir398c* **(c)** plants. The experiments were repeated at least three times with similar results, and data from one representative experiment are shown.

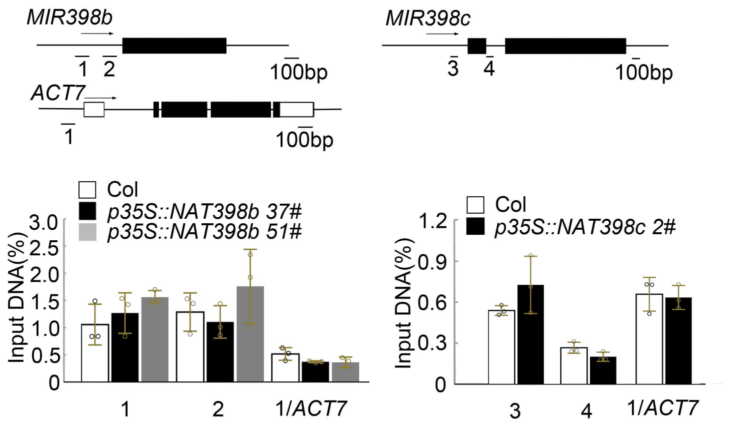

Supplementary Figure 8. ChIP analysis showing relative accumulation of RNA Pol II in *p35S::NAT398b* and *p35S::NAT398c* plants. Error bars are the mean ± SD. Significant differences were determined by one-tailed student's *t*-test (*, $P < 0.05$).

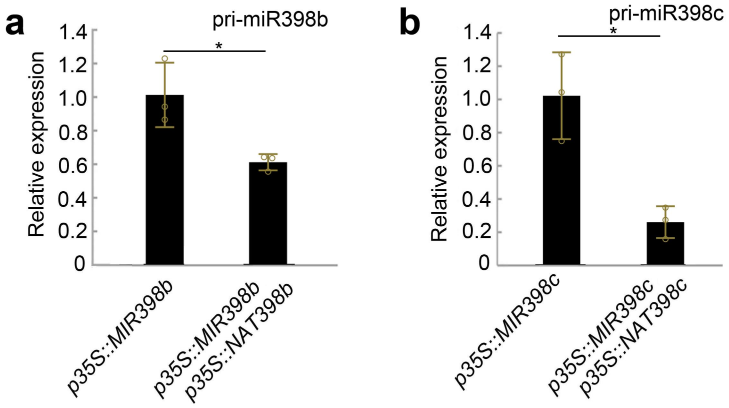

Supplementary Figure 9. Relative expression levels of *MIR398b/c* in Arabidopsis co-expressing *p35S::MIR398b/c* and *p35S::NAT398b/c* plants. **(a)** Relative expression levels of *MIR398b* in *p35S::MIR398b* and *p35S::MIR398b p35S::NAT398b* plants. **(b)** Relative expression levels of *MIR398c* in *p35S::MIR398c* and *p35S::MIR398c p35S::NAT398c* plants. Error bars are the mean ± SD. Significant differences were determined by one-tailed student's *t*-test (*, $P < 0.05$).

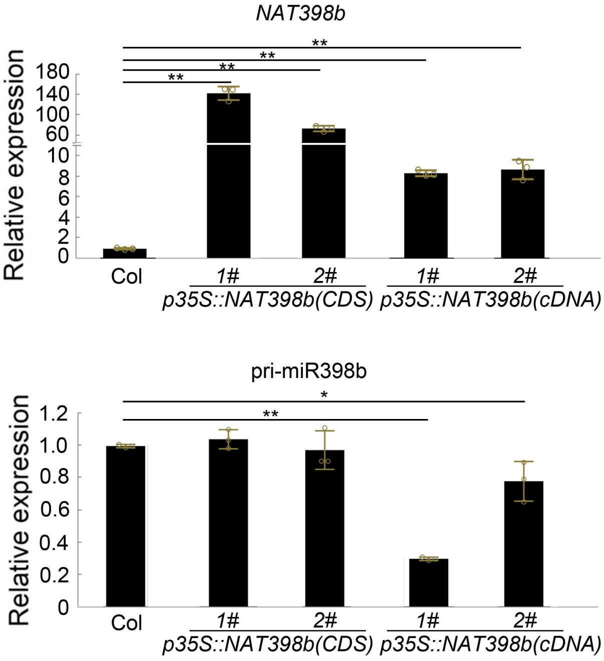

Supplementary Figure 10. Relative expression levels of *NAT398b* and pri-miR398b in transgenic plants. Error bars are the mean ± SD. Significant differences were determined by one-tailed student's *t*-test (*, $P < 0.05$; **, $P < 0.01$).

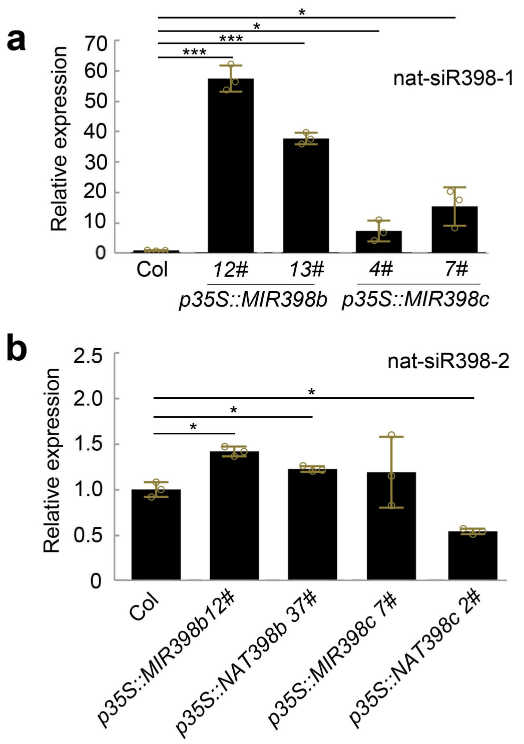

Supplementary Figure 11. Relative expression levels of nat-siR398-1 and nat-siR398-2 in transgenic plants. **(a–b)** Expression levels of nat-siR398-1 **(a)** and nat-siR398-2 **(b)** in the transgenic plants. Error bars are the mean ± SD. Significant differences were determined by one-tailed student's *t*-test (*, $P < 0.05$; **, $P < 0.01$; ***, $P < 0.001$).

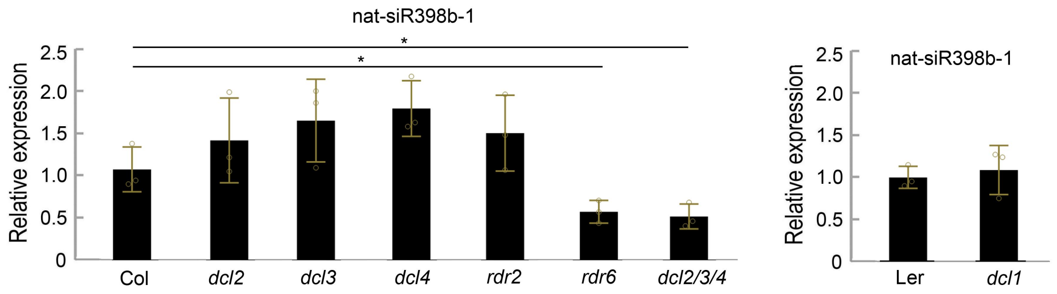

Supplementary Figure 12. Examination of nat-siR398b-1 accumulation in some small RNA biogenesis mutants. qRT-PCR showing nat-siR398b-1 accumulation. Error bars are the mean ± SD. Significant differences were determined by one-tailed student's *t*-test (*, $P < 0.05$).

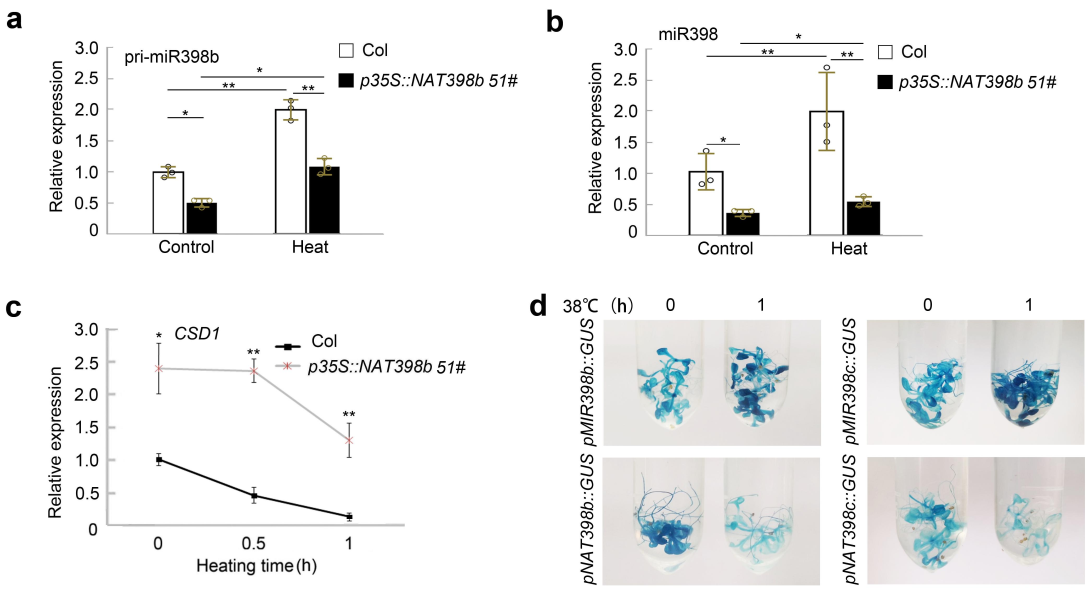

Supplementary Figure 13. Gene relative expression levels and GUS signals under heat stress. **(a–c)** Expression levels of *MIR398b* **(a)**, miR398 **(b)** and *CSD1* **(c)** in *p35S::NAT398b* plants under heat stress. **(d)** GUS staining of *pMIR398b/c::GUS* and *pNAT398b/c::GUS* plants exposed to heat stress. Error bars are the mean ± SD. Significant differences were determined by one-tailed student's *t*-test (*, *P* < 0.05; **, *P* < 0.01; ***, *P* < 0.001).

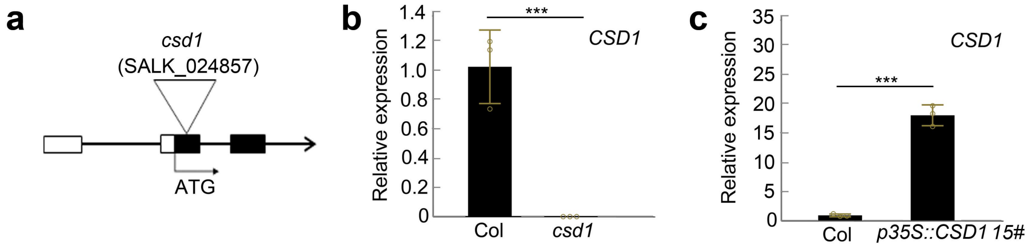

Supplementary Figure 14. Relative expression levels of *CSD1* in *csd1* and *p35S::CSD1* seedlings. **(a)** Diagram showing the location of the T-DNA insertion in the *csd1* mutant. **(b–c)** Relative expression levels of *CSD1* in *csd1* **(b)** and *p35S::CSD1* **(c)** seedlings. Error bars are the mean ± SD. Significant differences were determined by one-tailed student's *t*-test (*, $P < 0.05$; **, $P < 0.01$; ***, $P < 0.001$).

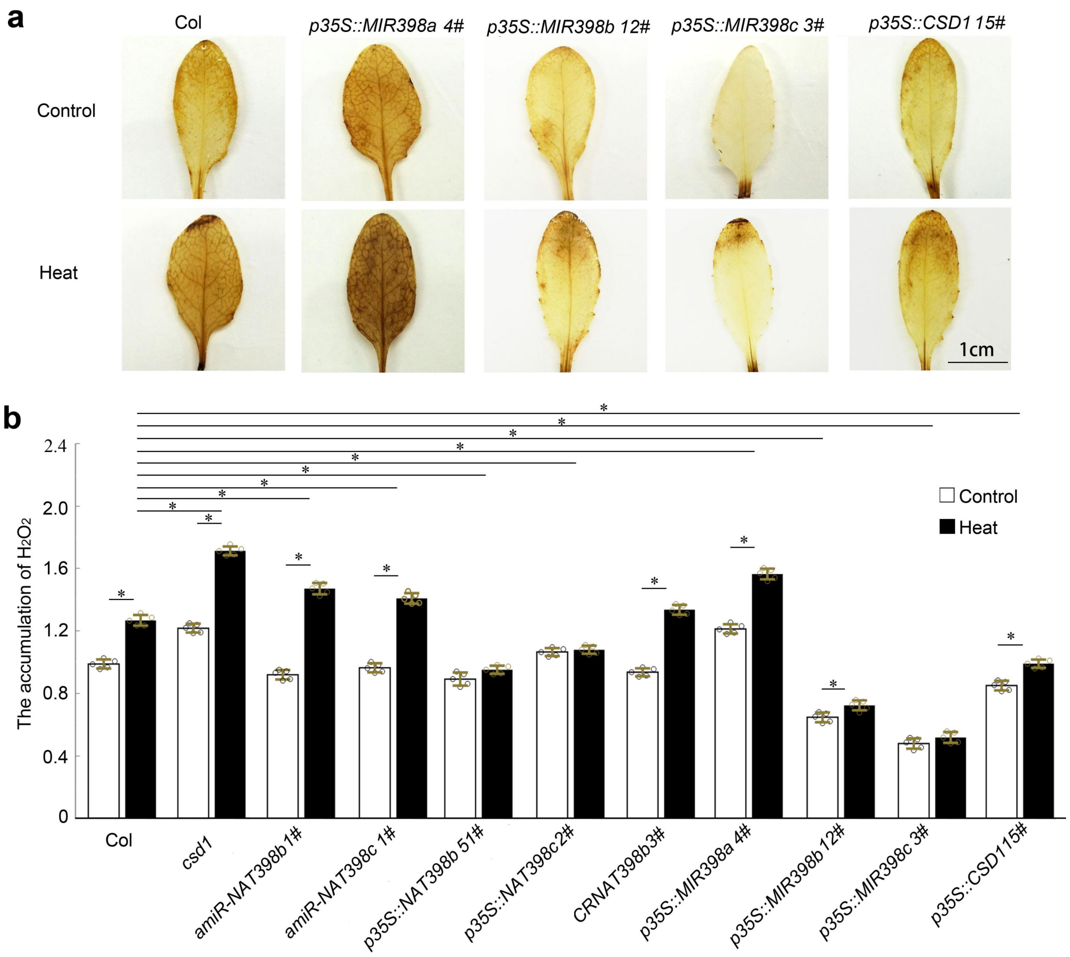

Supplementary Figure 15. H_2O_2 accumulation in transgenic plants. **(a)** DAB staining for H_2O_2 levels in transgenic and mutant plants. **(b)** The accumulation of H_2O_2 in transgenic plants. Error bars are the mean ± SD. $n = 5$ biologically independent samples. Significant differences were determined by one-tailed student's t-test (*, $P < 0.05$).

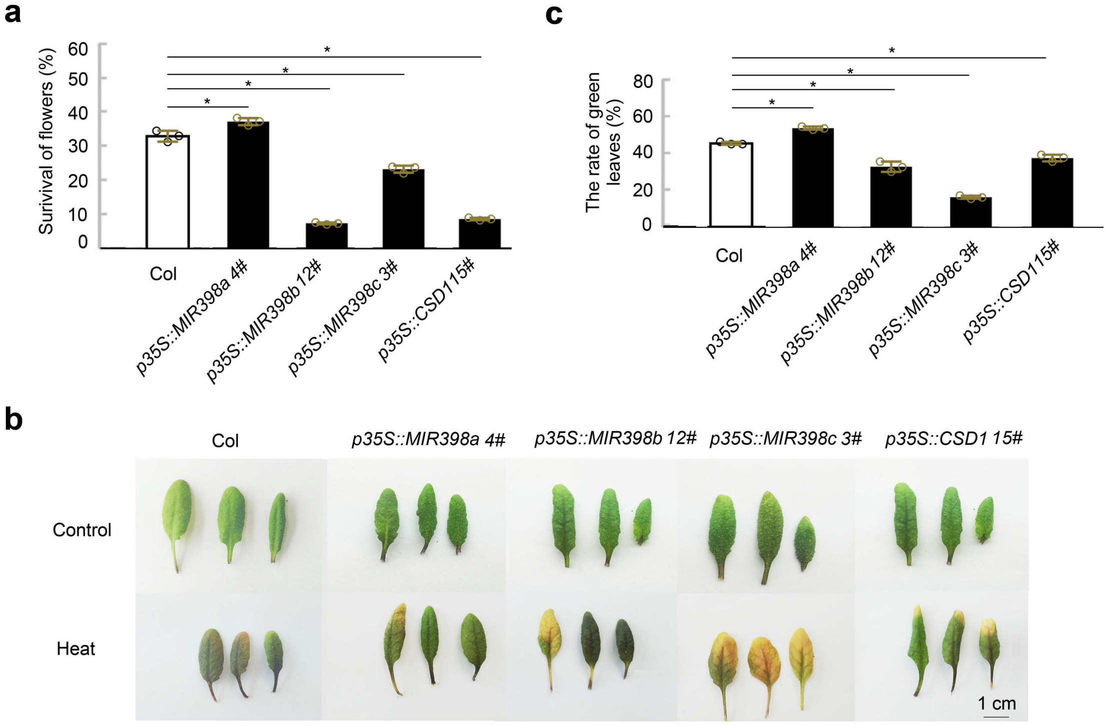

Supplementary Figure 16. Results of a thermotolerance assay of *p35S::MIR398a*, *p35S::MIR398b*, *p35S::MIR398c* and *p35S::CSD1* plants. **(a)** Survival rate of flowers of the wild-type, *p35S::MIR398a*, *p35S::MIR398b p35S::MIR398c* and *p35S::CSD1* plants under heat stress. **(b–c)** The phenotypes of detached leaves **(b)** and the rate of green leaves **(c)**. Error bars are the mean ± SD (*n* = 3 biological replicates, a replicate constitutes 60 biologically independent samples for **a**, and 12 biologically independent samples for **c**). Significant differences were determined by one-tailed student's *t*-test (*, *P* < 0.05).

Supplementary Tables

Supplementary Table 1. *cis*-NATs of *MIRNA* genes.

Plants species	MIRNA genes	cis-NATs	cis-NATs encode proteins
Arabidopsis thaliana	MIR5630a	AT1G33120	Yes
	MIR5630b	AT1G33140	Yes
	MIR5663	AT1G50440	Yes
	MIR156i	AT1G53160	Yes
	MIR5652	AT1G63130	Yes
	MIR5646	AT2G07680	Yes
	MIR156g	AT2G19420	Yes
	MIR5639	AT2G24340	Yes
	MIR836	AT2G25015	No
	MIR5021	AT2G28100	Yes
	MIR156j	AT2G42200	Yes
	MIR408	AT2G47020	Yes
	MIR171a	AT3G51380	Yes
	MIR826a	AT4G03038	No
	MIR401	AT4G08103	No
	MIR841a	AT4G13570	Yes
	MIR5661	AT4G19080	Yes
	MIR5634	AT4G30750	Yes
	MIR8178	AT4G38760	Yes
	MIR398b	AT5G14550	Yes
	MIR398c	AT5G14570	Yes
	MIR5631	AT5G20960	Yes
	MIR5657	AT5G27660	Yes
	MIR4243	AT5G48280	Yes
	MIR156h	AT5G55830	Yes
Brassica rapa	BrpMIR172a-1	Bra000498	Yes
	BrpMIR156h-1	Bra002873	Yes
	BrpMIR408a	Bra004482	Yes
	BrpMIR156j-2	Bra004674	Yes
	BrpMIR398b-1	Bra006261	Yes
	BrpMIR9563b	Bra007077	Yes
	BrpMIR398b-2	Bra008752	Yes
	BrpMIR5717	Bra009827	Yes
	BrpMIR171a-2	Bra012855	Yes
	BrpMIR9566	Bra013317	Yes
	BrpMIR156j-1	Bra016891	Yes
	BrpMIR9562	Bra022048	Yes

	BrpMIR167d	Bra023163	Yes
	BrpMIR162a	Bra028668	Yes
	BrpMIR1885a	Bra033746	Yes
	BrpMIR1885b	Bra033746	Yes
	BrpMIR9552a	Bra035208	Yes
	BrpMIR5630b-1	Bra035980	Yes
	BrpMIR171a-3	Bra036812	Yes
	BrpMIR156i	Bra039656	Yes
	BrpMIR5630b-2	Bra039999	Yes
	BrpMIR9552b	BrpMIR9552a	No

Supplementary Table 2. Reads of miR398 obtained by sRNA-seq.

	Replication 1	Replication 2
	CPM	CPM
miR398b/c	341.6728705	188.0862898
miR398a	0.076541858	0.045608897

Supplementary Table 3. miR398 accumulation in *csd1*, *p35S::NAT398b* and *p35S::MIR398a* plants compared with the wild type as ascertained by sRNA-seq.

Arabidopsis thaliana	Row.names	Fold Change (two biological replicates)
csd1 vs Col	ath-miR398a	1.020194572
	ath-miR398b/c	1.150632503
p35S::NAT398b vs Col	ath-miR398a	0.558572698
	ath-miR398b/c	0.633463593
p35S::MIR398a vs Col	ath-miR398a	1.280312028
	ath-miR398b/c	1.101038738

Supplementary Table 4. RACE results showing the relative accumulation of incorrect cleavage sites in 5′ single-stranded RNA regions of pri-miR398b.

Arabidopsis thaliana	Number at 5' SSR	*vs* Col
Col (n=40)	7	
p35S::amiR-NAT398b 3# (n=40)	5	-28.6%
p35S::NAT398b 37# (n=41)	14	+95.1%
p35S::NAT398b 51# (n=42)	12	+63.3%

Supplementary Table 5. Degradation products from pri-miR398b determined by sRNA-seq.

Arabidopsis thaliana	The rate of fragments (%)	*vs* Col
Col	7.65993603	
p35S::NAT398b	26.89663491	+251.13%
