# Supplementary material for: Natural antisense transcripts of MIR398 genes suppress microR398 processing and attenuate plant thermotolerance
Source: Nat Commun. 2020 Oct 22;11:5351. doi: 10.1038/s41467-020-19186-x (PMC7582911; doi:10.1038/s41467-020-19186-x)
Supplement: Supplementary file 3 — Descriptions of Additional Supplementary Files [file 41467_2020_19186_MOESM3_ESM.pdf]

Descriptions of Additional Supplementary Files

Supplementary Data 1

Description: Primer sequences used in this study. Supplementary Data 2. MIR398 and NAT398 genes in plants.
